# Music Therapy with Neonatal Intensive Care Unit-Discharged Mother-Infant Dyads: Developing a Method for Nurturing Communicative Parental Efficacy (CoPE with Music)

**DOI:** 10.3390/ijerph18168553

**Published:** 2021-08-13

**Authors:** Galit Calderon-Noy, Avi Gilboa

**Affiliations:** Music Department, Bar-Ilan University, Ramat Gan 5290002, Israel; avi.gilboa@biu.ac.il

**Keywords:** NICU, music therapy, dyadic therapy, self-efficacy, parental efficacy

## Abstract

While much advancement has been documented in the practice of music therapy in the neonatal intensive care unit (NICU) environment, there is currently a shortage of music therapy-based methods for NICU-discharged dyads. Back in their homes, mothers might feel alone, lacking guidance, and possibly losing their parental efficacy and their ability to communicate with their baby. In this article, we present a method for nurturing the communicative parental efficacy (CoPE) that was successfully practiced with several NICU-discharged dyads. In eight weekly sessions, the music therapist improvises with the dyad and focuses on (1) containing the mother’s emotions; (2) modeling musical interactions with the baby; and (3) practicing these musical interactions with the mother, enabling her to gain communicative parental efficacy. The basic ideas of CoPE are outlined, and a short case study is then described, to demonstrate how it is used. Finally, suggestions for future directions for the development of CoPE are provided.

## 1. Introduction

The biological maturation of an infant during pregnancy allows mothers to experience a parallel emotional process of maturation and preparation for parenthood [1]. In the case of preterm birth, however, the maturation process stops abruptly, and mothers of premature babies must face three main difficulties. Physically, recovery is required as a result of the complex birth [2]. Environmentally, adjustment to the threatening and paralyzing environment is needed [3], as the natural maternal care roles are taken abruptly by the medical staff [2]. Finally, emotionally, mothers need to cope with the trauma of preterm birth [4], the uncertainty surrounding their baby’s survival, and the feelings of fear, guilt, loss, and grief that might consequently be evoked [5]. Indeed, studies show that mothers experience the birth of premature babies as a crisis [6], and that they may grapple with feelings of sadness, depression [7], inability, incompetence, a sense of failure in caring for their babies [2], and guilt [8]. Sometimes these feelings undermine the mother’s mental state in the long run, and may also impair the infant’s development and the attachment between the mother and her infant [9]. This means that when a premature birth occurs, the infant’s physical condition and the mother’s physical–mental state will affect the quality of the initial attachment, and the mother may experience greater difficulty in acquiring control, proficiency, and self-efficacy in relation to parenting tasks [10].

Discharge from the neonatal intensive care unit (NICU) indicates that the mother and the baby are no longer in medical danger, but studies show that the coping challenges continue and, in some cases, even intensify. On the one hand, there may be relief, since the baby is no longer in danger and the mother has returned to a safe and familiar environment, but, on the other hand, there are increased feelings of parental stress and anxiety as a result of the new situation [7,11,12,13]. Zanardo, Fertoa, and Zacchelloa (2003) [14] found that the anxiety levels of mothers of preterm babies were higher at discharge than those of mothers of healthy infants. Upon arriving at home, mothers feel widespread uncertainty, medical concerns, and difficulty adapting to the new parenting situation [13]. Mothers are faced with the challenges of preterm care and may continue to experience distress as they become responsible for conducting procedures such as providing medication and developmental care, which are services that were previously provided by supportive healthcare professionals in a medical environment [15]. If the baby suffers from medical complications that affect its growth and development, this can be an additional source of stress for the parents [16], as well as the financial expenses that are involved in caring for the baby, and the financial losses caused by the lost work days [17,18]. Moreover, it was found that almost a third of mothers who give birth prematurely continue to experience a feeling of unresolved grief for up to nine months after the birth, indicating the persistence of trauma and stress, even after discharge from the NICU [19].

These persistent complex emotional states can consequently affect the mothers’ self-efficacy [20] and attachment patterns with their babies. Regarding self-efficacy, studies show that mothers who feel a high sense of self-efficacy in their functioning as a parent are likely to successfully establish a warm and harmonious relationship with their infants. In contrast, mothers with a low sense of parental self-efficacy are more likely to have difficulty coping [21]. This creates a vicious cycle, in which a low sense of parental self-efficacy leads to lower parental functioning, which leads to lower developmental outcomes, which, in turn, perpetuates and further exacerbates the low perception of parental self-efficacy, and so on [22]. The difficult feelings that accompany a mother after birth, and discharge from the NICU, might also affect the attachment patterns with the baby [23]. This can lead to impaired quality of care in preterm infants, and the negative effects may be reflected in the infant’s social development [24], as well as in the infant’s cognitive, emotional, and behavioral development [25].

Clearly, mothers and their babies need assistance not only during their hospitalization in the NICU, but also when they are already at home [26]. Although there are existing treatment options for mothers’ post-hospitalization, these are not as available as the treatments that are offered in NICUs, nor are they as prevalent. We will see that, especially in the field of music therapy, most treatment options focus on the NICU environment.

## 2. Existing Treatment Methods

In light of mothers’ needs post NICU discharge [11], various methods have been developed that focus on three main aspects. The methods that focus on environmental support provide a solution for parents, with regards to the preterm infant’s process of return, care, and physical adaptation in the home environment. This includes support groups with mothers who have experienced similar situations in the past [3], with medical staff who provide information, skills, and guidance for basic infant care [27,28,29], as well as follow-up and home visits that provide information regarding care and breastfeeding. In addition, support is provided by professionals, such as social workers and psychologists, who can help with identifying the signs of depression and anxiety [16]. The methods that focus on emotional support [27] include, among other things, active interventions that provide information and support in developing the parental role and improving parental self-efficacy [1]. The most effective type of support is when it is perceived by the mother as appropriate to her needs and requirements, and when it is provided in order to prevent the effects of stress from escalating [3]. Finally, the methods that focus on attachment support aim to bring together and strengthen the process of attachment between the mother and baby. Typically, in this type of treatment, the mother and baby are present together in the therapy room (dyadic treatment), which allows for “real-time” observation of their relationship and exploring possibilities to improve the communication between them [30].

The field of music therapy also developed treatment options to support mothers who gave birth prematurely, and they cover all three aspects that are described above, although most of these methods focus on treatment within the NICU [31,32,33,34,35,36,37,38]. However, there is currently a shortage of music therapy-based methods that care for mothers and preterm infants, and address their psychosocial needs post-NICU hospitalization [39,40].

There are some examples of the effective use of music therapy with mothers and their babies, as a dyad or in a group of dyads, not specifically intended for post-NICU hospitalization [41,42,43,44,45,46,47,48]. They use different music therapy-oriented techniques that can be adapted to work with post-NICU dyads. For instance, music therapy groups have been developed, with the aim of providing social support to parents and ways of coping with parenting challenges, such as the Sing & Grow treatment method [41,42,49]. This method was developed to enhance daily parenting skills, and to strengthen the attachment of at-risk families using various musical activities, such as instrumental play, action songs, and movement songs, to encourage physical touch and bonding. The Music and Movement program [48], to give another example, uses vocal, gestural, and rhythmical movement activities to promote the interactions of first-time mothers with their 2- to 6-month-old infants, and to enhance maternal well-being. Another form of group therapy that is intended for first-time mothers is Baker and Mackinlay’s (2006) [50] educative music therapy program, in which mothers are trained to use lullabies effectively with their very young babies (6 weeks to 18 months). Other music therapists focused their work on at-risk mothers. Pasiali (2014) [51] worked with mothers who were suffering from depression, who lacked the emotional sensitivity that their babies needed, and she showed how practicing parenting through singing to the baby can strengthen the mother–infant bond. Hojsak and Katušić (2020) [52] implemented creative and Orff music therapy for mothers at risk and their 1- to 4-month-old babies, and showed that this enhanced their engagement with their babies and improved their parenting abilities (and see also, Reilly et al., 2018 [53], who reported successful work with mothers suffering from postpartum depression). Taken together, much of this knowledge can be adapted and examined with post-NICU dyads, to see if they are effective in promoting communication and attachment.

Music therapy programs that are intended specifically for NICU-discharged dyads are, to date, quite scarce [40]. Palazzi et al. (2020) [54] reported of their work with a mother and her preterm baby, during their NICU hospitalization and 4 months after their discharge. This treatment, called “The Music Therapy Intervention for the Mother-Preterm Infant Dyad (MUSIP)”, aims to support the mother singing to her baby, in the NICU, and then at home. Observations of the dyad showed that MUSIP empowered the mother’s competence to interact with her baby, and facilitated dyadic synchrony. Another project that focuses on NICU/discharged dyads is the “longSTEP” project [40,55,56]. In this ongoing project, conducted in Argentina, Colombia, Israel, Norway, and Poland, the effect of music therapy in the NICU and/or post discharge is concurrently tested on dyads of premature babies and their parents, after 6, 12, and 24 months. However, this project does not suggest a specific procedure or protocol to be used systematically after discharge, as we would like to suggest here.

In summary, although music therapy can offer many unique contributions to the rehabilitation and development of the mother–baby attachment, and to the mother’s parental efficacy, not enough focus has been directed specifically to post-NICU dyads [40]. Taking into account that much of the interaction between mothers and babies is of a musical nature [57,58,59], and that the stressful conditions of preterm birth and treatment in the NICU can jeopardize the development of this musical communication, it is reasonable to assume that music therapy can be a suitable and effective medium by which to treat this situation. Such a method should attempt to rehabilitate the communicational abilities of the dyad and the efficacy of the mother, using the “language” that mothers and babies know best, which is musical in essence. We will now describe the CoPE method, which has been developed over the past few years.

## 3. CoPE with Music—An Evolving Method

In the past few years, I (the first author of this article) have been working with post-hospitalized mothers and their preterm babies, in a dyadic context, and felt that the communication between the mother and baby was damaged. For instance, the natural sing-song vocality of the mother was significantly diminished and, in some cases, totally absent, and it seemed that mothers were not emotionally connected to the vocal signs of their babies. It was as if the detaching effects of the incubator and the hospital environment continued to influence communication, even at home, and, as time went on, instead of improving, the mother’s parental efficacy deteriorated.

Self-efficacy is a term that was developed by Bandura [60], and it refers to people’s belief in their ability to successfully complete a task. Later, researchers developed the term parenting self-efficacy, to define parents’ confidence in their ability to successfully manage parental challenges [22]. Here, we use a more specific term, communicative parental efficacy, to indicate the mother’s ability to communicate with her baby, and, more importantly, her belief in her ability to create and maintain such communication. As mentioned above, I could see that if mothers did not receive help with their babies, upon returning home, their parental efficacy, and especially their communicative parental efficacy, would continue to diminish. I could also see how music therapy techniques could be applied to this situation, focusing on restoring the mother’s communicative parental efficacy through music therapy.

After working with seven dyads, for a period of two years, I felt that, indeed, the musical techniques I was using, and the method that evolved, was very effective, and that in a relatively short time, usually less than 10 sessions, mothers were able to restore their maternal intuitive abilities, and the dyad started to function much more smoothly. I felt that the method that I developed in the clinic needed to be further established and examined systematically. To do this, I conducted, as part of my MA thesis, two dyadic case studies of post-NICU hospitalized mothers and their preterm babies, and I thoroughly documented and analyzed them according to the qualitative case study framework. A case study research offers a qualitative approach to explore a case, or several cases, in a real-life context or setting, while allowing a comprehensive and thorough collection of data through various information sources (observations, audiovisual data, interviews, reports, and documents), and to produce a rich case description and themes. This research aims to offer an in-depth understanding of the case and the phenomena, as a means to understanding other cases, and how they represent and affect the studied population [61].

Two mothers of first-born preterm babies, one of them at the age of 3 weeks gestational age (GA—the age the child would be had the pregnancy reached full term (40 weeks)) and the other at the age of 12 weeks GA, were contacted through publication in relevant websites, and they offered to join the study. They each met me for eight 90-min weekly sessions with their babies, in a well-equipped room at the Bar-Ilan University music therapy clinic. The first session was an intake to get to know the mother and her baby, and their history since pregnancy, through birth, hospitalization in the NICU, and discharge. The eighth and last session was a summary of the treatment process and a follow-up of the mother’s account of her state after the treatment, compared to the beginning of the treatment. All the sessions were audio recorded, transcribed, and subjected to content analysis [62], which was used to identify the meaningful categories. Musical material, such as songs and vocalizations, were notated and analyzed. The study was approved by the ethics committee of the music department at Bar-Ilan University. The mothers were assured that they could receive the treatment even if they decided to leave the study, and that their confidentiality would be strictly protected.

In the framework of the current article, the focus will mainly be on the description of the method that was developed. However, it can also be briefly reported here that the treatment was very meaningful and effective. The mothers who participated in the study were interviewed before and after the treatment, and their qualitative data were gathered in categories that indicated that they felt improvement in several aspects. The turbulent feelings they had towards themselves, their babies, and the environment, were relieved, leaving them with much more energy to develop good communication with their babies. The method described here, named CoPE with music (CoPE stands for communicative parental efficacy), is based on the first author’s accumulated research findings and clinical experience with post-NICU dyads. We will first describe the basic ideas that constitute the CoPE method, and then, to demonstrate how it is used, we will describe the work with one of the dyads, treated as part of the first author’s MA thesis.

## 4. CoPE with Music—Fundamentals

CoPE with music is a short-term treatment, consisting of eight sessions. This is due to my experience with mothers of preterm infants and their busy schedules with their babies, and sometimes with various medical procedures. Consequently, they find it difficult to commit to long-term treatments. In addition, we aimed to provide the mother with solid tools as quickly as possible, and to give her the feeling that she was capable of implementing these tools independently, outside of the treatment. Short-term treatment, with a known and fixed number of sessions, is, therefore, more suitable than long-term treatment. Indeed, my pilot study that was based on pre-post in-depth interviews with the mothers, showed that improvement in parental efficiency and communication with the baby could be achieved in a relatively short amount of time, and that eight sessions were enough to get the dyad back on track.

Each session is 90 min long, which is a relatively lengthy treatment session. My experience showed that once the mothers and their babies leave home, they need as much quality time as possible. With a tiny baby, everything can be time consuming, such as getting to the clinic, getting organized in the clinic with the stroller, feeding the baby if necessary, and it is important to provide the conditions to do everything calmly. I also noticed that when the mothers were in the clinic, especially in the first sessions, they required a considerable amount of time to ventilate and to express themselves. Ninety-minute sessions enabled this to be an integral part of the session, before proceeding to other activities, such as working with the baby.

The clinic is set to create a calm and supportive environment, comparable to a nest. It is large enough for the three of us and for a stroller. There is a beanbag placed in one corner, which can be very useful if the baby needs to be breast or bottle fed, and it is useful for some of the dyadic musical activities. There are also chairs, in case the mother feels more comfortable (physically and/or mentally) to interact in an upright posture. In addition, there is a hot beverage station and some light refreshments in the room, in case the mother is hungry or thirsty. I found that when they are at home, many mothers are exceedingly occupied with caring for their babies, and they neglect to take care of their most basic physical needs. In such cases, it is important that food and drink are available in the room, so that this issue can be raised and worked through.

The clinic is equipped with a modest variety of instruments that are light and transportable, easy to play intuitively, with gentle sound production. Some examples are small bells, a small xylophone, a cylinder chime, cluster bells, a slide whistle, and a small 14 ocean drum. Maracas and a tambourine are also important, as they can play a role in encouraging the baby’s motion and movement (i.e., by offering the instrument for the baby to grab, each movement is echoed with sound, which then can reinforce additional motion, see case study). These instruments are generally not expensive, so the mother may purchase them if she wants to continue musical activities at home, after the treatment ends. A monochord, although bigger and more expensive, is also suggested, because it can provide a soothing and calming sound bath that can be valuable in the dyadic context. Small instruments that can be swallowed, and instruments that cannot be cleaned easily, should be avoided.

There are the following three modes of intervention that take place in the treatment process: (1) Containment, in which the music therapist provides space and time to hear the mother, her problems, and her pains. Much of this mode of intervention takes place verbally. The mother speaks about the traumatic event, the premature birth, and she ventilates, while the music therapist listens, contains, and builds intimacy, trust, and a rapport with the mother. Musical interventions, such as jointly listening to a song that is significant to the mother and reflecting on it, can enable further processing of difficult moments that the mother experienced during her pregnancy, the birth, or the hospitalization at the NICU. This mode of intervention can obviously take place only if the baby is asleep or calm. If the baby is either upset or playful, attention will naturally drift to interacting with him or her; (2) Modelling, in which the music therapist suggests how to musically interact with the baby and demonstrates this to the mother. Of course, this can happen only once enough trust has been built and both the mother and baby are comfortable with such an interaction. Typical interventions in this mode can be calming the baby down when he or she is upset, voicing the baby’s expressions when he or she is playful, or cuddling the baby and singing a song to him or her. While such activities might seem basic and even instinctual to many mothers, I find that mothers of preterm babies, due to the non-communicative side effects of the hospitalization period, need someone to demonstrate how this is done, and to then practice it themselves. For many mothers, this is their first opportunity to experience their baby in a close and intimate light, to understand their physical, emotional, behavioral, and cognitive state, and to respond accordingly. Needless to say, many of these interactions are musical in nature, such as vocal mirroring, echoing, use of musical instruments to expand attention and motion span, and cuddling and singing to the baby; (3) Practicing, in which the mother tries to interact with her baby according to what the music therapist demonstrated to her. The music therapist observes, acknowledges, encourages the dyad, and offers adjustments when required. This mode of interaction is usually possible when the mother has ventilated enough and experienced enough containment, and once she felt she could independently implement what the music therapist modeled. However, once the mother is ready for this mode of intervention, it can become very prominent in the treatment and important for the mothers. Often, it is here that she feels that she is regaining her communicative parental efficacy. Frequently, she will report that at home, too, she feels increasingly more capable of dealing with her baby, using the musical interventions that she practiced in the treatment sessions.

The flow of the session depends on the dyad, and on its immediate needs. If, for instance, the mother walks into the session and the baby is asleep, it is a good opportunity to catch up on the mother’s wellbeing, and to see whether she needs any emotional support and containment (the containment mode of intervention). If, then, the baby wakes up crying, it becomes a good opportunity to model to the mother how to soothe the baby (the modeling mode of intervention), or for the mother to practice soothing her baby (the practicing mode of intervention). The music therapist does her utmost to follow the natural flow of the dyad, and to assist, according to what is required at each point, and not to dictate anything. This way, the dyad can feel that the clinic is almost an extension of their daily life routine, yet it offers additional options for learning, growing, and experimenting, in a non-judgmental environment.

Although much of the knowledge and the techniques that are offered in CoPE with music have been introduced in the past, the uniqueness of the method is that it is specifically tailored for NICU-discharged dyads. As such, it takes into account the typical needs and requirements of the mother, who needs to regain her parental efficacy, the baby, who needs the conditions to close developmental gaps, and the dyad that needs to develop communication abilities. These needs are met by various music therapy techniques, some adopted from developmental-oriented music therapy (e.g., encouraging the mother to echo her baby’s vocalizations, etc.), and others from the NICU setting (e.g., the choice of meditative and ‘soft toned’ musical instruments). CoPE with music integrates these techniques and provides a coherent method that makes it relevant and effective, specifically for NICU-discharged dyads. It is also the combination of environmental, emotional, and attachment supportive aspects in one framework that makes this method unique.

## 5. CoPE with Music—A Case Study

Rina (pseudonym) is a 28-year-old married mother to Itamar (pseudonym), her firstborn son who was born prematurely at week 31 GA. Our first session was mostly dedicated to getting acquainted with Rina and learning about the premature birth of Itamar. The session had the characteristics of an informative intake session, but obviously it provided Rina with a tremendous amount of emotional ventilation that was not possible for her outside the room. Rina shared her challenging experiences, from labor through hospitalization in the NICU to post discharge. Immediately after birth, her baby was taken from her to intensive care, and she first saw him only 14 h later. She described these moments as follows: “*It’s weird, there’s an empty feeling, I had something here* (points to her stomach) *and then I didn’t*.” During the time the baby was in the incubator, she had a hard time connecting with him, as is clear from the following: “*You don’t feel the natural mothering experience that usually starts the second the baby’s born*”. She said she was probably under the influence of some “defense mechanism” preventing the emotional attachment to her baby, to avoid the risk of depression that would occur if the baby did not survive. She also felt that the control over her baby was taken from her, as follows: “*Suddenly he’s not only yours, you also have to share him with the entire staff*”. When she did have the opportunity to take care of him, it was an emotional challenge, as shown by the following: “*It took me quite a while to realize that he’s mine, this “thing” there–that it needs me*”. Upon discharge from the NICU, as she arrived at an empty house, without the 24/7 support and care, and without her husband, who was absent for days due to his intensive work, she felt incapable, scared, and very lonely, as she explained with the following: “*I was used to being supported by everyone around, and all of a sudden I come home, I’m alone, and if I’m doing something wrong-it’s all my responsibility*”.

**Figure 1:**
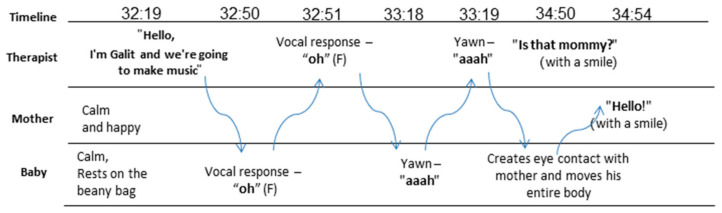
An illustration of a mother–baby music therapist vocal interaction.

As we move along to the beginning of the second session, Rina entered the clinic while Itamar was asleep, so I decided to start with her. She told me about a song, titled *You Are Your Mother’s Hero*, that helped her to deal with the difficulties and challenges that she experienced in the NICU. Although she always loved this song, in the NICU it took on a special meaning for her, and, at times, she found herself singing it to her baby. This did not happen when he was still in the incubator, but only when he was placed on her, “skin-to-skin”, she said the following: “*You suddenly feel like you’re interacting with a human being, not with a plastic box.*” Ten minutes into the session, Itamar woke up, and we immediately turned our attention to him. He was vocalizing a bit, so I spontaneously echoed him and initiated a “conversation” out of it. Figure 1 shows how this conversation unfolded, and how it began between me and Itamar (minute 32:30), eventually pulling Rina in as well (minute 34:54). I explained to Rina that I was using all my senses to try to understand what Itamar was “saying”, in terms of his vocalizations, his movements, his facial expressions, any rhythm he formed, or possible interactions he was making with his environment.

After a while, Itamar started to cry and I used this opportunity to demonstrate how music can help. I invited Rina to seek tiny nuances that could make him irritable, for example, examining the basic external (environmental) and internal stimuli pertaining to him (clothing, fatigue, soiled diaper, hunger) and, after eliminating all of them, I invited her to pick him up, hum quietly, and just experience being together. For Rina, Itamar’s crying was a stressful event and she felt she was not capable of controlling it. The technique I showed her was simple and possible to implement, so it had the potential of making Rina realize that she could do it, and that she could start gaining communicative parental efficacy.

**Figure 2:**
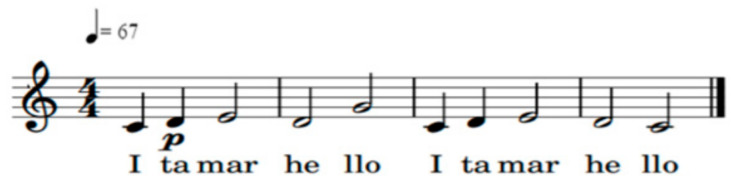
Notes of the “Hello song”.

In the third session, when Rina came in, Itamar was crying. Similarly to the previous session, this was a good opportunity to demonstrate how music could be used to soothe him. I first verified that there were no external or internal stimuli in the room that could distract Itamar, and then I sang a “Hello song” (see notes in Figure 2). I sang repeatedly, so that Rina and Itamar could learn the song. I sang very softly, at a constant pace, and stopped at the end of each musical sentence. I used excessive mimicry and I inserted Itamar’s name in the song numerous times.

As the song continued, Itamar stopped crying and became very focused on me. After I finished singing, his body moved excitedly and he seemed to be pleased, perhaps asking us to continue the song. I explained to Rina what I was doing all along and afterwards. Itamar was now calm, so the conversation from our last session with Rina about the *You Are Your Mother’s Hero* song could continue. We listened to the song from my YouTube playlist and Rina said that the phrase “Let me breathe you in, just a little more” from the song, was especially significant to her when she was hospitalized. Together, we could see why, after the premature birth and in the NICU, she needed more time to adjust and to process what was happening to her. When Itamar became restless again, I started practicing, with Rina, how to handle the situation on her own, with the following steps: first check what his needs might be, and, if appropriate, try using a song to soothe him. Rina started singing a familiar song to Itamar. Although the song she chose was too intense for the situation, with too many words and without pauses, and even though she sang much too quickly and too loudly, it was important that she let her voice be heard, and that she felt comfortable enough to sing to Itamar out loud. This was an important first step in making her voice a key component in her development towards communicative efficacy.

In the fourth session, Rina told me how, following her successful vocal experience in the third session, she started to use more singing at home and even outside the home. Excitedly, she recounted how she started developing an awareness to Itamar’s subtle signs, she said the following: “*He has several types of communication. He starts making these voices and expects a response. When I respond, he mimics the voice or gives a hint as to what he wants next. He plays with his voice, sometimes high pitched and sometimes low, and usually he really enjoys it.*” She went on, with the following, to tell me how she managed to soothe Itamar with music: “*When he’s in a nervous state, he usually doesn’t like it when I talk to him. He only likes when I sing, and especially songs that he likes, it calms him down.*” The highlight was when she took Itamar to get his vaccinations, and how she sang to him during and after the vaccination, to calm him (and herself). We were both amazed to see how naturally she caught on and how quickly she implemented what she experienced in the clinic. The modeling and the practice in the previous session were very effective in prompting Rina to action.

Another issue that came up in this session was Rina’s feeling that she was constantly criticized by others. For example, with regards to her approach to Itamar’s crying. While she thought it was important to let him cry before picking him up, others in her family thought this was not good mothering. I gave Rina room to express and process these feelings, and this eventually gave her more confidence with her opinions and to withstand criticism. Here, too, Rina’s voice was empowered, and with it, her parental efficacy. As we progressed, other emotional issues came up, and talking about them seemed to free Rina. She said that her openness to her emotions, particularly the difficult ones, was influencing her husband as well. Only now did he start expressing his emotions, and revealing how difficult these past months, in and out of the NICU, were.

As the therapeutic process progressed to the second half of the treatment (sessions five to eight), the impact of vocal musicking expanded from the dyadic mother–infant cycle to the immediate family. For example, Rina brought a video of her grandmother singing to Itamar, and told me how once when she could not soothe Itamar, her husband reminded her of the following: “*why don’t you just sing him his song?*”, and indeed, within two minutes of singing, Itamar fell asleep.

I noticed that when Rina sang, she barely took the time to breath and for silence, as if she had to continuously fill the void with sounds and words. When I shared this with her, she agreed that “*…usually I’m used to a faster rhythm with Itamar, there’s never a dull moment. But here, when we pause or do nothing, I can see that he’s actually fine… he focuses, he’s thinking about something, and I don’t necessarily have to overwhelm him with words and things to do.*” Rina associated this with her need to constantly be in control of things, and especially in limiting the expression of negative emotions, such as anger, frustration, and sadness. She recalled that she was brought up in a home where such emotions were not legitimate, and she now understands that this was reflected in her parenting style. After this insightful session, she allowed herself to get upset at home and feel uncomfortable. The world that Rina came from was structured and planned, and it allowed her to always feel confident, stable, and in control of the situation. Now, for the first time, she relinquished the structure and criticism from her surroundings, and began to adapt to what suited her at that moment. As a result, she said that she felt a lot less pressure. It enabled her to better understand Itamar’s world and the connection between them, and it also enabled her to gain confidence as a mother and to confront external criticism, as follows: “*I began to break free of the advice that people kept giving me*”.

**Figure 3:**
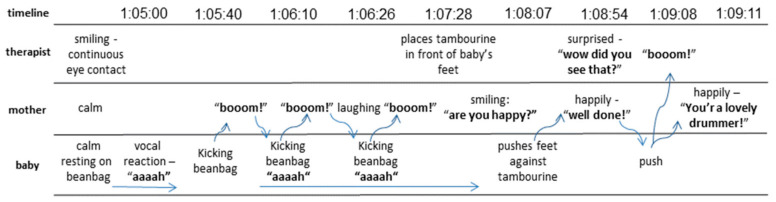
An illustration of a music and movement improvisation.

About that time, Rina started to improvise vocally with Itamar. One such improvisation is illustrated in Figure 3. In this instance, Itamar was lying comfortably on the beanbag and, as he began to kick, I suggested that Rina place her hand close to his feet and that every time he kicked, she would make a synchronized sound (she chose “boom!”). After this worked very nicely, we placed the tambourine near Itamar’s feet, so that each time he kicked, he got immediate auditory feedback for his action. This too turned out to be a very enjoyable game. Itamar seemed to have fun, and Rina felt confident enough to replace me.

In the seventh session, Rina, aware that the treatment was coming to an end, asked to learn more ways to musically interact with Itamar. I suggested that she listen to his heartbeat and develop an improvisation around this. This required her to place her head close to Itamar’s chest and to listen carefully to his heartbeat. There is a lot of intimacy in this position, and a need to mindfully “let the silence speak”. It also counteracted the negative experiences that Rina might have had in the NICU, where Itamar’s heartbeat was accompanied by loud and stressful monitor sounds. Rina experimented with this and noticed that Itamar’s heartbeat was different to hers. I then suggested that she played the drum in rhythm with Itamar’s heartbeat. She liked this very much and concluded the following: “*we took his natural rhythm, which he feels inside, and gave it a place in the world outside.*” We talked more about rhythm and discovered that Rina experienced the rhythm of her life as hectic, and that she never managed to accomplish what she planned to. She then raised the idea that she tried to change this pace, and adopt some of Itamar’s pace, which she perceived as much calmer.

In the eighth and final session, we summarized the treatment. Rina described the significant change that she experienced in her relationship with Itamar. At the beginning of the process, she found it difficult to relate to Itamar, and she felt no feedback from him, “*like walking in the dark*”. Now, Rina felt as if she understood Itamar, and that she saw how important he was to her and how much she meant to him. Rina felt a huge improvement in terms of dealing with Itamar’s day-to-day care. She said that throughout the sessions, she received practical tools to identify and interpret Itamar’s reactions and to soothe him, as shown by the following: “*Today I am much more capable of attending to him.*” Rina also recounted that she gained musical and vocal instruments that helped her to communicate with Itamar and enabled a broader understanding of his world, as outlined by the following: “*Today I can sing him his songs and I see that it does affect him… he gives me feedback about what he likes and what he doesn’t like*”. Rina also felt that, through CoPE, she developed personally. She gave herself more credit to feel negative emotions and to process them, as follows: “*I used to ignore the negative feelings I felt. But since we talked, I give my emotions more freedom.*” This was possible because of the containing atmosphere in the room, as understood from the following: “*I felt I had a place to say everything I want… everything I feel.*” Finally, she learned to filter out criticism, and to rely on her intuition much more confidently, and speak her voice.

## 6. Discussion

CoPE with music is a method that is tailored specifically for NICU-discharged dyads. It is designed to restore the mother’s parental efficacy and the dyadic communication abilities that are often negatively affected by preterm birth and a stressful stay in the NICU. In this article, we demonstrate that music is central in promoting these goals, and that giving the mother simple musical tools to connect with her baby can make a significant difference in a short-term intervention. The mother’s voice, which may have been blocked out in the non-intimate and stressful atmosphere of the NICU, is especially important in this process. It is not only her concrete voice that she learns to reconnect to and to use lovingly with her baby, but also her metaphorical voice that she learns to feel confident about, and to express out loud to her environment. This is gained not only through technical counsel of what to do and how (modeling and practicing modes of intervention). CoPE with music also includes a very important supportive emotional component, in which the mother is invited to express her innermost feelings without being judged or criticized (the containing mode of the intervention). Thus, she has the opportunity to decipher her emotions during the stressful and traumatic period that she experienced in the NICU and after discharge, and how they might have affected the relationship with her baby. Through the emotional component, other issues, such as parenting styles and family member assistance, can be raised and developed to the benefit of the evolving mother–baby relationship. Indeed, we saw that CoPE with music affected not only the mother and the baby in the room, but that it could be applied to day-to-day life and that its effects influenced other family members as well (e.g., father, grandmother).

CoPE with music is consistent with many of the recommendations for practice that are suggested by Bieleninik et al. (2020) [40]. As suggested by these authors, it is important to provide music therapy treatment only when required and in an appropriate location and frequency. CoPE with music was practiced until now on a weekly basis for 8 weeks in a clinic, but considering that some might need different settings, it is advisable to keep on experimenting with other possible versions of CoPE with music. It is our understanding, however, that mothers need assistance more than they are willing to admit, and that being out of the home environment, although technically less convenient, has the potential to provide a quiet and neutral environment that is essential for a fresh start. CoPE with music is also consistent with Bieleninik et al.’s (2020) [40] recommendation to aim for a variety of goals to be addressed in treatment (e.g., interaction between family members, communication, bonding, play skills, sensory stimulation, regulating mothers’ moods), and to be flexible with the goals that are chosen for a specific dyad. Music has so much to offer, and it can be shaped in so many different ways to suit the situation and the specific dyad, and so, the more flexible, creative, and playful the music therapist is, the better. Bieleninik et al. (2020) [40] also point out that post-NICU, mothers might feel lonely, and that they no longer have staff members to talk to; therefore, it is important that music therapists do not hesitate to use verbal interventions, and to lend a listening ear to the mothers. Indeed, in CoPE with music, the containing mode of intervention is mostly based on talking and listening, and the other modes of intervention are also accompanied by verbal guidance.

CoPE with music is still in its infancy, with only a pilot study completed. Similarly to a newborn, it needs attention and care to develop and to thrive. We, therefore, recommend that CoPE with music is further implemented, explored, and refined. It is important to know how to develop and improve it, whether it requires adjustments for different dyads, in different environments, and whether it can be implemented in different family settings, including the father and perhaps other family members as well.

## 7. Conclusions

NICU-discharged dyads are in a vulnerable situation and there is a need to develop methods tailored to helping them. In this article, we present the fundamentals of such an evolving music therapy-based method (CoPE with music), and a case study to demonstrate how the method is implemented. Through this case, it could be observed how in eight 90 min sessions, the mother gained parental efficacy, and how her communication with her baby improved. The unique combination that the music therapist offered in these sessions, both that of being a person whom the mother can trust to ventilate with, and that of being a guide and a model for communication with her baby, is probably what enabled this rapid shift. In this case, it can also be observed how the musical solutions that were offered by the music therapist, were at the core of the change, and how the mother and her baby became so much more musical towards each other. Once this musicality was substantiated, communicational parental efficacy started to evolve and unfold, to the point that the treatment could be terminated after less than two months.
